# Minocycline Regulates PARP-1 and HDAC3 Pathways to Inhibit Inflammation and Oxidative Stress in LPS-Induced Acute Lung Injury

**DOI:** 10.5812/ijpr-161381

**Published:** 2025-06-29

**Authors:** Chao Luo, Xixi Zhu, Jiefei Liang, Chunyan Zhu, Guohua Shen, Weibin Wu

**Affiliations:** 1 Department of Medicine and Health, Shaoxing Key Laboratory of Targeted Drug Delivery and Targeted Materials, Shaoxing University Yuanpei College, Shaoxing, China; 2 School of Basic Medicine, Zhaoqing Medical University, Zhaoqing, China; 3 Affiliated Hospital of Shaoxing University, Shaoxing, China

**Keywords:** Minocycline, Acute Lung Injury, Alveolar Epithelial Cells, PARP-1, HDAC3

## Abstract

**Background:**

Acute lung injury (ALI) is characterized by excessive lung inflammation and apoptosis of alveolar epithelial cells, resulting in acute hypoxemic respiratory failure. Minocycline, a tetracycline antibiotic, is known to have excellent anti-inflammatory activity.

**Objectives:**

The present study aims to reveal the protective effect and potential mechanism of the anti-inflammatory effects of minocycline on lipopolysaccharide (LPS)-induced ALI in mice and A549 cells.

**Methods:**

We investigated the role of minocycline in ALI mice and inflammation-induced damage to alveolar epithelial cells using various experimental approaches, including histological staining, enzyme-linked immunosorbent assay (ELISA), quantitative real-time PCR, flow cytometry, western blot analysis, and other relevant assays.

**Results:**

Pre-treatment with minocycline effectively attenuated LPS-induced ALI in vivo by inhibiting inflammation and oxidative damage, improving pathological changes in the lungs, alleviating pulmonary edema and protein exudation, and suppressing neutrophil aggregation. In vitro, minocycline suppressed the inflammatory response of human alveolar epithelial A549 cells, as evidenced by the inhibition of inflammatory cytokine and oxidative damage biomarker expression, reduction in intracellular reactive oxygen species (ROS) production, alleviation of mitochondrial damage, and inhibition of cell apoptosis. Subsequent mechanistic studies revealed that the protective effects of minocycline against ALI may be attributed to its suppression of poly (ADP-ribose) polymerase-1 (PARP-1) and histone deacetylase 3 (HDAC3) expression.

**Conclusions:**

In conclusion, our study presents minocycline as a potential candidate for ALI therapy and provides an experimental foundation for investigating its anti-inflammatory mechanisms in the treatment of ALI. Further therapeutic value awaits verification in clinical and preclinical studies.

## 1. Background

Acute lung injury (ALI) and its most severe manifestation, acute respiratory distress syndrome (ARDS), are life-threatening lung diseases characterized by an excessive inflammatory response and increased alveolocapillary permeability, leading to alveolar edema, hypoxemia, and respiratory failure (1). The key event in the pathogenesis of ALI/ARDS is the excessive activation of the inflammatory response, which involves infiltration of inflammatory cells, alveolar-capillary destruction, and excessive oxidative stress characterized by aberrant release of reactive oxygen species (ROS) and alveolar epithelial cell apoptosis (2, 3). Recent findings indicate that inflammatory injury and extensive apoptosis of pulmonary alveolar type II epithelial cells are important causes of impaired epithelial barrier function in ALI (4). The overproduction of pro-inflammatory cytokines and chemokines, such as tumor necrosis factor (TNF)-α, interleukin (IL)-1β, IL-6, IL-8, and intercellular adhesion molecule (ICAM)-1, can impair epithelial cells and promote recruitment and activation of inflammatory cells. The activated neutrophils further aggravate pulmonary injury (5-7). Thus, attenuation of inflammation and oxidative stress may be an effective treatment for ALI/ARDS.

Poly (ADP-ribose) polymerase-1 (PARP-1) is the best understood member of the PARP family, playing an important role in DNA repair, chromatin remodeling, transcription, and regulation of the cell cycle (8). In the last decade, several studies have demonstrated the role of PARP-1 in lung inflammation, such as in asthma and ALI (9). Poly (ADP-ribose) polymerase-1 suppression by genetic deletion or pharmaceutical inhibitors has been shown to reduce inflammatory cell recruitment to the airways in lipopolysaccharide (LPS)-induced murine pulmonary inflammation (10). Various studies have shown that PARP-1 influences the expression of inflammatory genes via interaction with transcription factors [such as nuclear factor kappa-B (NF-κB)], thereby regulating the expression of cytokines, chemokines, and adhesion molecules, and playing a critical role in the inflammatory cycle (8-10).

Histone deacetylase 3 (HDAC3), an essential chromatin-modifying protein within the histone deacetylase superfamily, plays a pivotal role in regulating diverse transcription factors associated with inflammatory responses and related diseases (11). Studies have confirmed that HDAC3 expression is upregulated in various inflammatory conditions, and selective HDAC3 inhibition modulates inflammation in multiple pathologies (12). Therefore, targeting HDAC3 to regulate inflammatory mediators and responses holds great promise for the effective treatment of inflammatory diseases.

Minocycline is a tetracycline antibiotic with potent antimicrobial activity. In addition to its antibacterial properties, a large body of research has shown that minocycline possesses anti-inflammatory and cytoprotective properties (13, 14). The anti-inflammatory effect of minocycline is mainly mediated by inhibition of PARP-1 (15). Furthermore, our molecular docking studies reveal that minocycline exhibits strong binding affinity to HDAC3.

## 2. Objectives

Therefore, we hypothesize that minocycline may protect alveolar epithelial cells in ALI by alleviating the inflammatory response through inhibition of PARP-1 and HDAC3. In the present study, we conduct both in vitro and in vivo experiments to evaluate the ability of minocycline to protect against LPS-induced ALI damage. Furthermore, we explore the mechanisms underlying this protective effect. The results of these experiments suggest that minocycline is a potential candidate for ALI therapy and provide an experimental foundation for investigating its anti-inflammatory mechanism in the treatment of ALI.

## 3. Methods

### 3.1. Materials and Reagents

Minocycline was purchased from Dalian Meilun Biotechnology Co., Ltd. (Dalian, China). The LPS was purchased from Sigma-Aldrich. Mouse IL-1β, IL-6, and TNF-α enzyme-linked immunosorbent assay (ELISA) kits were purchased from Dakewe Biotech Co., Ltd. (Shenzhen, China). Reactive oxygen species, myeloperoxidase (MPO), superoxide dismutase (SOD), malonaldehyde (MDA), and glutathione peroxidase (GSH-Px) assay kits, JC-1 mitochondrial membrane potential detection kit, Hematoxylin and Eosin Staining kit, trizol, and BeyoFast^TM^ SYBR Green qPCR mix were purchased from Beyotime Biotechnology (Shanghai, China). Primers and probes of tested genes were synthesized by Sangon Biotech Co., Ltd. (Shanghai, China). Specific antibodies for PARP-1, NF-κB p65, p-p65, IκB kinase β, p-IKKβ, inhibitor of kappa B (IκBα), p-IκBα, nuclear factor erythroid 2-related factor 2 (Nrf2), heme oxygenase-1 (HO-1), B-cell lymphoma-2 (Bcl-2), Bcl-2 associated X protein (Bax), cleaved-Caspase3, HDAC3, and β-actin were purchased from Affinity Biosciences (OH, USA).

### 3.2. Murine Model of Lipopolysaccharide-Induced Acute Lung Injury

Male BALB/c mice (6 - 8 weeks old), weighing 20 - 25 g, were purchased from the Guangdong Medical Laboratory Animal Center (Guangzhou, China). All animal experimental procedures were approved by the Ethics Committee for Animal Experimentation of Zhaoqing Medical College (2024-018), performed in compliance with the ARRIVE guidelines, and carried out in accordance with the National Institutes of Health Guide for the Care and Use of Laboratory Animals. The mice were housed at room temperature (25 ± 2℃) under a 12 h light/dark cycle and 40 - 50% relative humidity, with unlimited access to food and water. Mice were randomly assigned to four groups (n = 6 per group): The control group, LPS group, LPS + minocycline (10 mg/kg) group, and LPS + minocycline (30 mg/kg) group. All groups were orally administered their respective treatments for 3 consecutive days. The control and LPS groups received PBS, while the treatment groups received 10 mg/kg or 30 mg/kg minocycline. Twenty-four hours after the final administration, the LPS and minocycline groups received intranasal delivery of 50 μL LPS (40 μg/mL), while the control group received PBS. To minimize pain, mice were humanely euthanized via cervical dislocation following intraperitoneal injection of pentobarbital sodium after treatment completion. Lung tissues were then collected for further analysis.

### 3.3. Lung Wet/Dry Weight Measurement

The lung wet/dry (W/D) ratio was used to assess the severity of pulmonary edema. After sacrifice, left lung tissues were immediately weighed (wet weight, W), then dried at 48℃ for 72 h to obtain the dry weight (D). The W/D ratio was then calculated.

### 3.4. Histopathology

Lung tissues were fixed in 10% formalin for 48 h, embedded in paraffin, sectioned at 5 μm thickness, and stained with Hematoxylin and Eosin (H&E). Lung injury scores were assessed according to a standard protocol (16).

### 3.5. Cellular Analysis of Bronchoalveolar Lavage Fluid

After anesthesia and euthanasia, a tracheostomy was performed, and a cannula was inserted into the trachea. The lungs were lavaged three times with ice-cold PBS, totaling 1.5 mL. A 0.1 mL sample was used for total cell count with a hemocytometer. The remainder was centrifuged at 500 g for 10 min at 4℃. Total protein in the bronchoalveolar lavage fluid (BALF) supernatant was quantified using the BCA method, with bovine serum albumin as a standard. The supernatant was aliquoted and stored at -70℃. The pellet was resuspended in PBS and used for differential cell counts with Wright-Giemsa-stained smears.

### 3.6. Enzyme-Linked Immunosorbent Assay

Cytokine levels (IL-1β, IL-6, and TNF-α) in BALF were measured using ELISA kits, following the manufacturer’s instructions.

### 3.7. Measurement of Oxidative Stress Biomarkers and Myeloperoxidase Activity

Lung samples were homogenized and centrifuged at 3000 rpm at 4℃ for 10 min to collect the supernatant. Myeloperoxidase, SOD, and MDA activities were measured using respective detection kits per the manufacturer’s instructions. Total protein content in the supernatants was determined using the BCA assay kit.

### 3.8. Cell Culture and Treatment

Human pulmonary epithelial A549 cells were obtained from the American Type Culture Collection (ATCC, Manassas, VA, USA). Cells were cultured in RPMI-1640 medium with 10% fetal bovine serum and 2 mM L-glutamine, in a humidified incubator at 37℃ with 5% CO_2_. A549 cells were seeded in 12-well plates at a density of 1×10⁶ cells/mL and treated with LPS (10 μg/mL) and minocycline (0, 10, or 30 μg/mL) for 24 h.

### 3.9. Real-time qPCR

Total RNA was extracted from treated A549 cells using trizol reagent and reverse-transcribed into cDNA using the BeyoRT^TM^ III cDNA synthesis kit (with gDNA EZeraser) from Beyotime. RT-PCR was performed using BeyoFast^TM^ SYBR Green qPCR mix on a QuantStudio 5 real-time PCR system (Applied Biosystems, USA). Primer sequences are listed in Table 1. Relative gene expression was analyzed using the ΔΔCt method.

**Table 1.** Sequences of RT-PCR Primers

Primers	Sequence（5’-3’）	Length/bp
**GAPDH**		197
F	GGAGCGAGATCCCTCCAAAAT	
R	GGCTGTTGTCATACTTCTCATGG	
**TNF-α**		124
F	AGCTGGTGGTGCCATCAGAGG	
R	TGGTAGGAGACGGCGATGCG	
**IL-1β**		85
F	GCCAGTGAAATGATGGCTTATT	
R	AGGAGCACTTCATCTGTTTAGG	
**IL-6**		149
F	ACTCACCTCTTCAGAACGAATTG	
R	CCATCTTTGGAAGGTTCAGGTTG	
**IL-8**		194
F	TTTTGCCAAGGAGTGCTAAAGA	
R	AACCCTCTGCACCCAGTTTTC	
**ICAM-1**		80
F	TGCAAGAAGATAGCCAACCAAT	
R	GTACACGGTGAGGAAGGTTTTA	

Abbreviations: TNF-α, tumor necrosis factor-α; IL-1β, interleukin-1β; IL-6, interleukin-6; IL-8, interleukin-8; ICAM-1, intercellular cell adhesion molecule-1.

### 3.10. Reactive Oxygen Species Activity Assay

Cells were treated according to the experimental procedure, then incubated with 20 μmol/L DCHF-DA for 30 min at 37℃ in the dark. Reactive oxygen species levels were visualized via fluorescence microscopy (excitation 485 nm, emission 535 nm).

### 3.11. Evaluation of Mitochondrial Membrane Potential

Mitochondrial membrane potential (MMP) was assessed using a JC-1 detection kit. Cells were incubated with 5 μg/mL JC-1 staining solution at 37℃ for 20 min, then rinsed twice with PBS. The MMP was evaluated using a fluorescence microscope (excitation 488 nm), based on the red/green fluorescence intensity ratio. A decrease in this ratio indicates mitochondrial depolarization.

### 3.12. Cell Apoptosis Analysis

Cells were cultured in 6-well plates and processed per the experimental protocol. After harvesting, cells were washed with PBS and stained with Annexin V-FITC and 10 μL propidium iodide (PI) (Beyotime, China). Apoptosis was assessed by flow cytometry using the FITC Annexin V Apoptosis Detection Kit I (Beyotime, China), following the manufacturer's instructions.

### 3.13. Western Blot

Total protein from treated cells was extracted using RIPA lysis buffer supplemented with PMSF (1 mM) and a phosphatase inhibitor cocktail (Beyotime, China). Protein concentration was determined using a BCA assay kit. Equal amounts of protein were separated by 10% SDS-PAGE and transferred to Immobilon-E PVDF membranes (Merck, Germany). Membranes were blocked with 5% non-fat milk for 1 h at room temperature and incubated overnight at 4℃ with primary antibodies (all 1:1000). After washing, membranes were incubated with HRP-conjugated secondary antibodies (1:5000, Affinity Biosciences, OH, USA). Protein bands were visualized using an electrochemiluminescence (ECL) detection system.

### 3.14. Statistical Analysis

Statistical analysis was performed using SPSS version 19.0. Data are presented as mean ± standard deviation (mean ± SD). One-way ANOVA and *t*-tests were used to analyze the data. A P-value < 0.05 was considered statistically significant.

### 3.15. Molecular Docking

The 3D molecular structure of minocycline was retrieved from the PubChem database (PubChem CID: 164676) and saved in .sdf format. The 3D protein structures of PARP-1 and HDAC3 were obtained from the RCSB Protein Data Bank (PDB ID: 5DS3) and saved in .PDB format. Molecular docking was conducted using the CB-Dock2 server (https://cadd.labshare.cn/cb-dock2/index.php).

## 4. Results

### 4.1. Minocycline Protects Against Lipopolysaccharide-Induced Acute Lung Injury In Vivo

**Figure 1.**
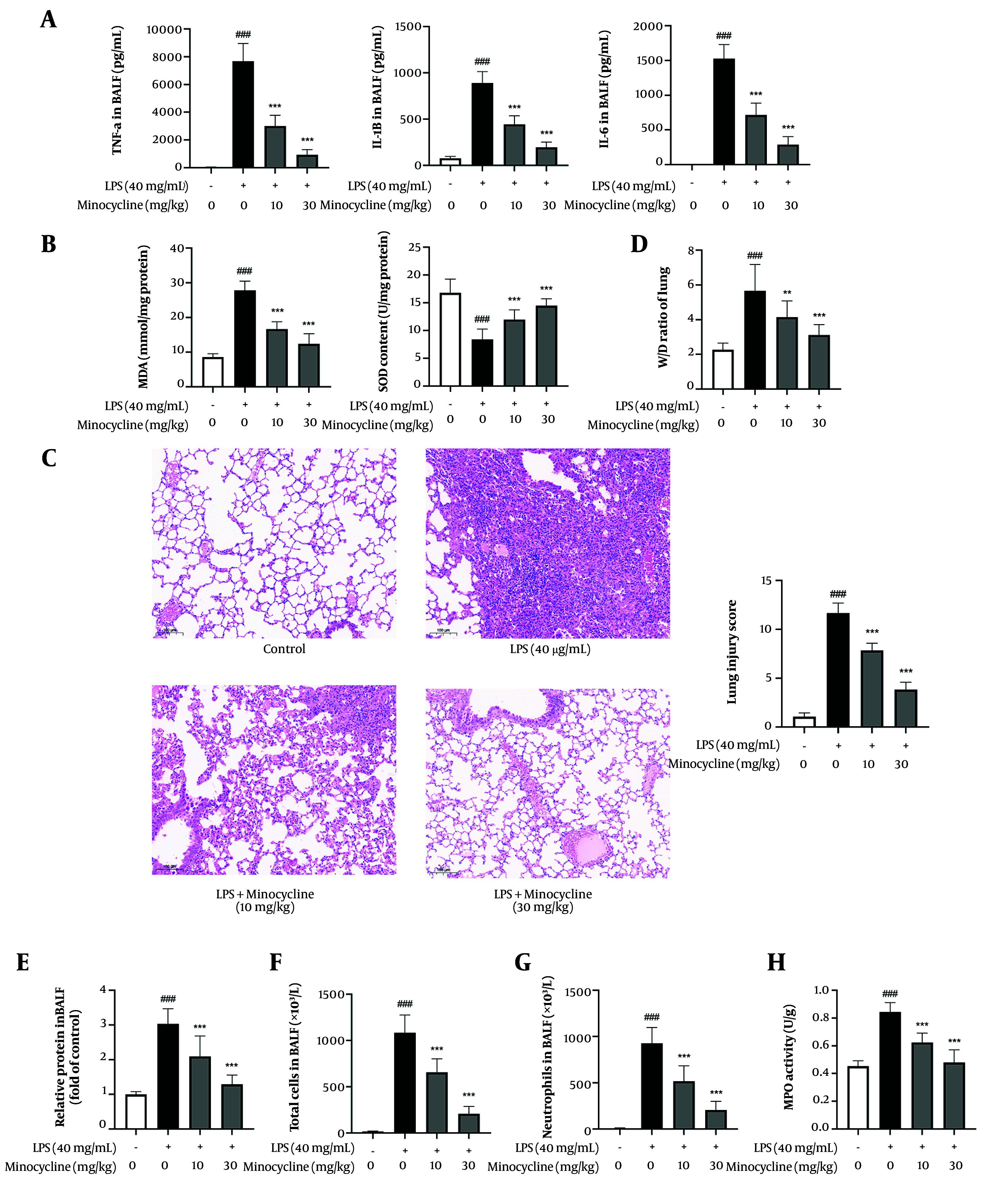
Minocycline reduces lung inflammation caused by lipopolysaccharide (LPS) in vivo. A, proinflammatory cytokines level in bronchoalveolar lavage fluid (BALF) [tumor necrosis factor (TNF)-α, interleukin-1β (IL-1β), IL-6]; B, biomarkers of oxidative stress [malonaldehyde (MDA), superoxide dismutase (SOD)] of lung tissues; C, representative results of Hematoxylin and Eosin (H&E) staining and pathology scores of lung tissues (scale = 100 μm); D, wet/dry weight ratio of lungs; E, relative protein content; F, total cells; and G, neutrophils in BALF; H, MPO activity of lung tissues [(n = 6) values are mean ± SD. ### P < 0.001 vs. the control group, ** P < 0.01, *** P < 0.001 vs. LPS-treated group.

To assess the therapeutic potential of minocycline in ALI, an LPS-induced ALI murine model was established. Minocycline treatment at 10 mg/kg and 30 mg/kg significantly reduced the expression of pro-inflammatory cytokines (TNF-α, IL-1β, IL-6) and markers of oxidative stress (MDA, SOD) in lung tissues (Figure 1A and B). Histopathological analysis revealed that LPS induced severe lung injury characterized by interstitial edema, hemorrhage, alveolar wall thickening, and increased inflammatory cell infiltration. Minocycline treatment markedly attenuated these histological changes in a dose-dependent manner (Figure 1C). 

Consistently, the lung wet/dry (W/D) weight ratio indicated that minocycline reduced pulmonary edema and improved alveolar permeability (Figure 1D). Additionally, minocycline significantly decreased total protein concentration in BALF (Figure 1E) and the number of infiltrated cells (Figure 1F), further confirming its protective role against pulmonary vascular leakage. Since neutrophil infiltration is a hallmark of ALI, MPO activity and neutrophil counts in BALF were assessed (17). Minocycline markedly suppressed neutrophil recruitment and MPO activity (Figure 1G and H), highlighting its anti-inflammatory potential in ALI.

Collectively, these findings demonstrate that minocycline effectively alleviates LPS-induced lung injury and inflammation (detailed P-values are provided in Appendix 2 in Supplementary File).

### 4.2. Minocycline Attenuates Lipopolysaccharide-Induced Inflammation and Oxidative Stress in A549 Cells

**Figure 2.**
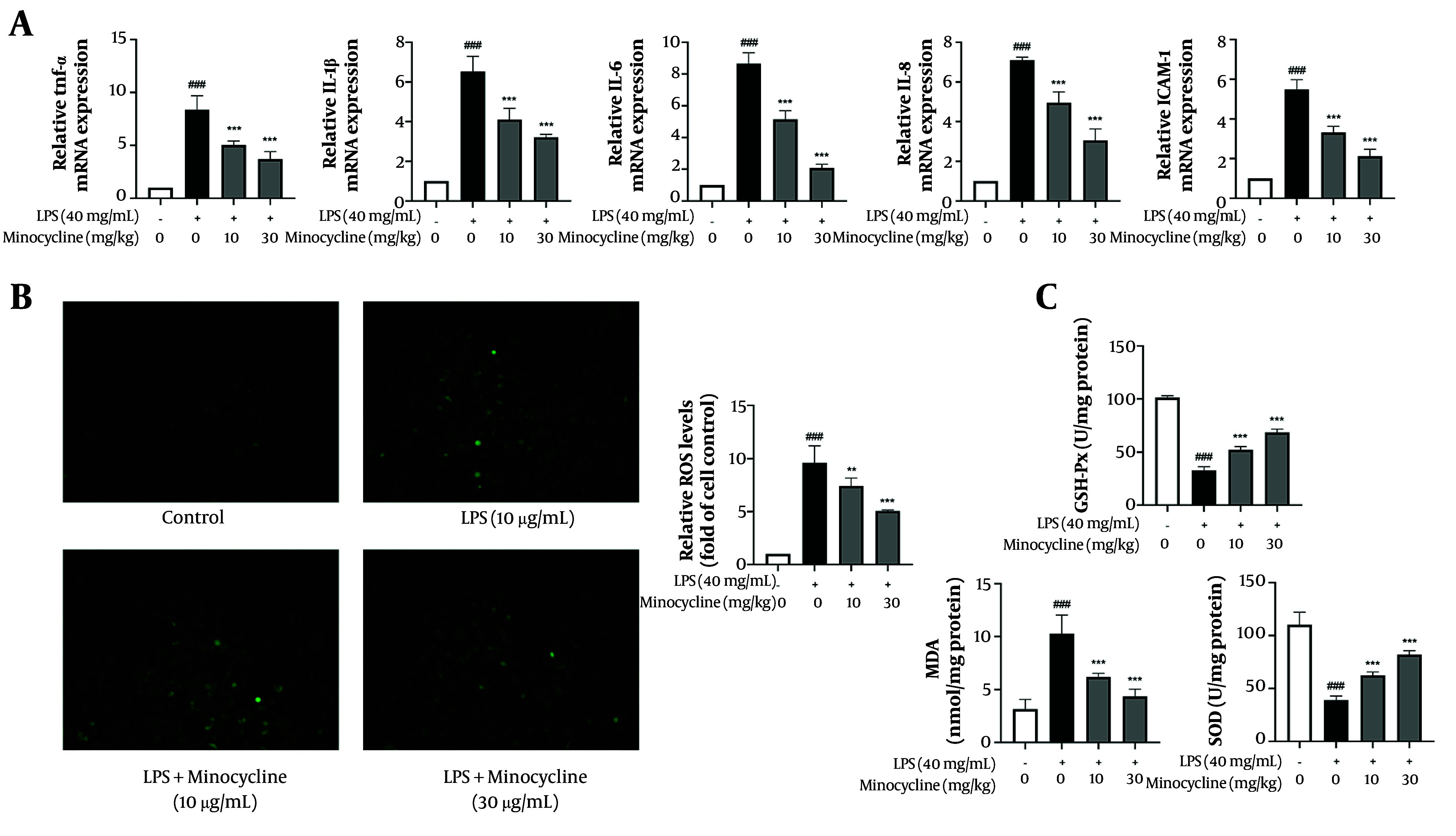
Minocycline inhibits inflammation and oxidative damage in lipopolysaccharide (LPS)-induced A549 cells. A, the mRNA levels of tumor necrosis factor (TNF)-α, interleukin-1β (IL-1β), IL-6, IL-8, and intercellular adhesion molecule (ICAM)-1 in LPS-induced A549 cells; B, intracellular reactive oxygen species (ROS) level; and C, biomarkers of oxidative stress [malonaldehyde (MDA), superoxide dismutase (SOD), glutathione peroxidase (GSH-Px)] in LPS-induced A549 cells [(n = 6) values are mean ± SD; ### P < 0.001 vs. the control group, ** P < 0.01, *** P < 0.001 vs. LPS-treated group.

As alveolar epithelial cells serve as frontline responders to pulmonary insults, we used LPS-stimulated A549 cells to mimic epithelial inflammation in ALI. Treatment with minocycline (10 and 30 μg/mL) significantly suppressed the mRNA expression of pro-inflammatory mediators including TNF-α, IL-1β, IL-6, IL-8, and ICAM-1 (Figure 2A). Moreover, minocycline mitigated oxidative stress by reducing ROS generation, MDA, and SOD levels, while enhancing GSH-Px activity (Figure 2B and C).

These results suggest that minocycline protects alveolar epithelial cells from LPS-induced inflammatory and oxidative damage, supporting its therapeutic relevance in ALI (exact P-values are listed in Appendix 3 in Supplementary File).

### 4.3. Minocycline Inhibits Lipopolysaccharide-Induced Apoptosis and Mitochondrial Dysfunction in A549 Cells

**Figure 3.**
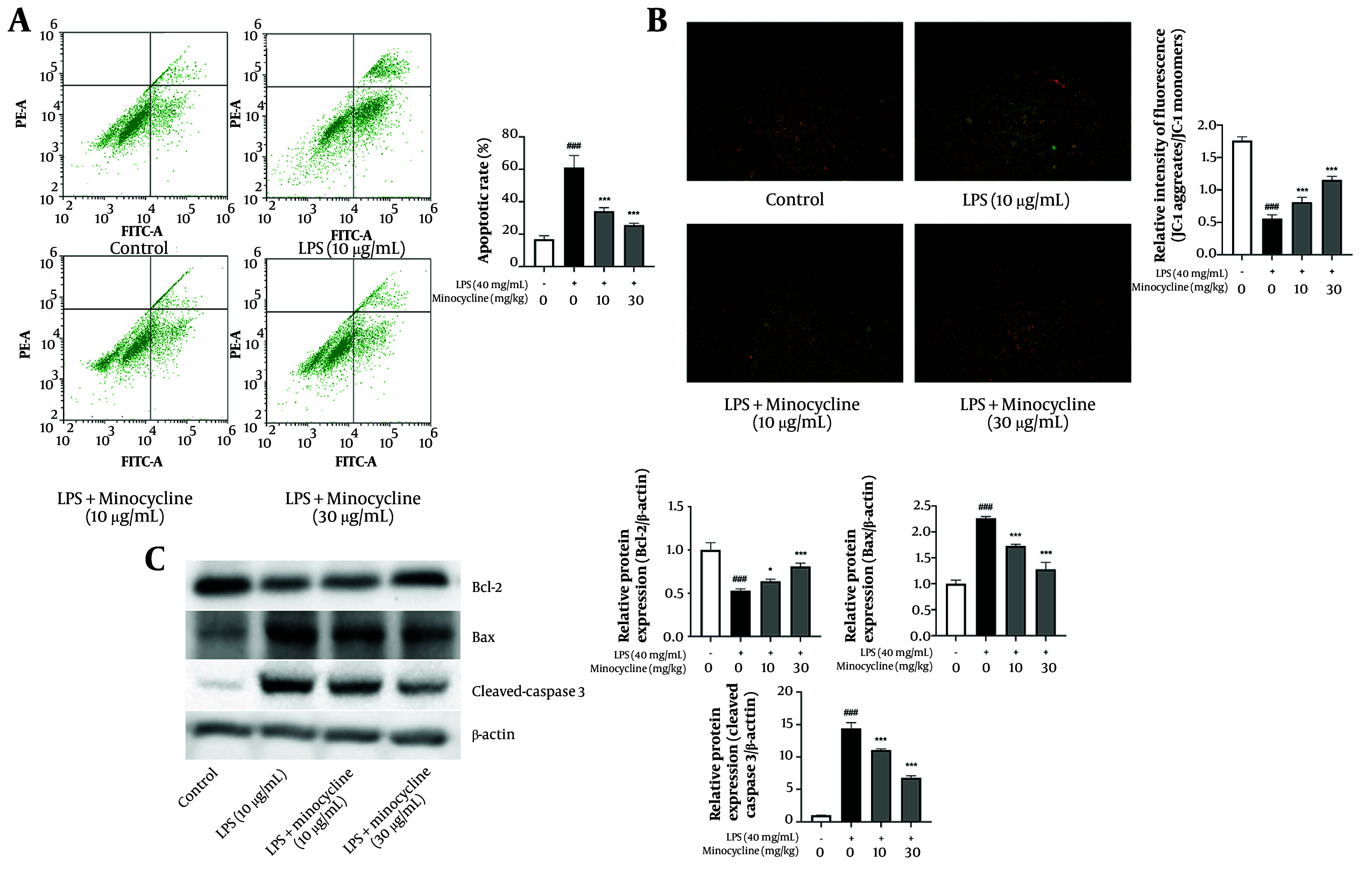
Minocycline inhibits the apoptosis and mitochondrial damage in lipopolysaccharide (LPS)-induced A549 cells. A, flow cytometry analysis for cell apoptosis in lipopolysaccharide (LPS)-induced A549 cells; B, the mitochondrial membrane potential which indicates by the red/green fluorescence ratio was measured by the JC-1; C, the B-cell lymphoma-2 (Bcl-2), Bcl-2 associated X protein (Bax), and cleaved-Caspase3 expression of LPS-induced A549 cells [(n = 6) values are mean ± SD; ### P < 0.001 vs. the control group, * P < 0.05, *** P < 0.001 vs. LPS-treated group..

Flow cytometry analysis revealed a significant increase in apoptosis in A549 cells following LPS stimulation, which was effectively reversed by minocycline treatment (Figure 3A). JC-1 staining demonstrated that LPS induced mitochondrial depolarization, as evidenced by a shift from red to green fluorescence (18). Minocycline significantly restored mitochondrial membrane potential, indicating protection against mitochondrial injury (Figure 3B). 

At the molecular level, minocycline upregulated the anti-apoptotic protein Bcl-2 and downregulated pro-apoptotic Bax and cleaved-Caspase 3, suggesting that its protective effect involves the inhibition of mitochondrial-mediated apoptosis pathways (Figure 3C) (detailed P-values are provided in Appendix 4 in Supplementary File).

### 4.4. Minocycline Reduces Lipopolysaccharide-Induced Activation of Poly (ADP-ribose) Polymerase-1 and Histone Deacetylase 3 Signaling Pathways

**Figure 4.**
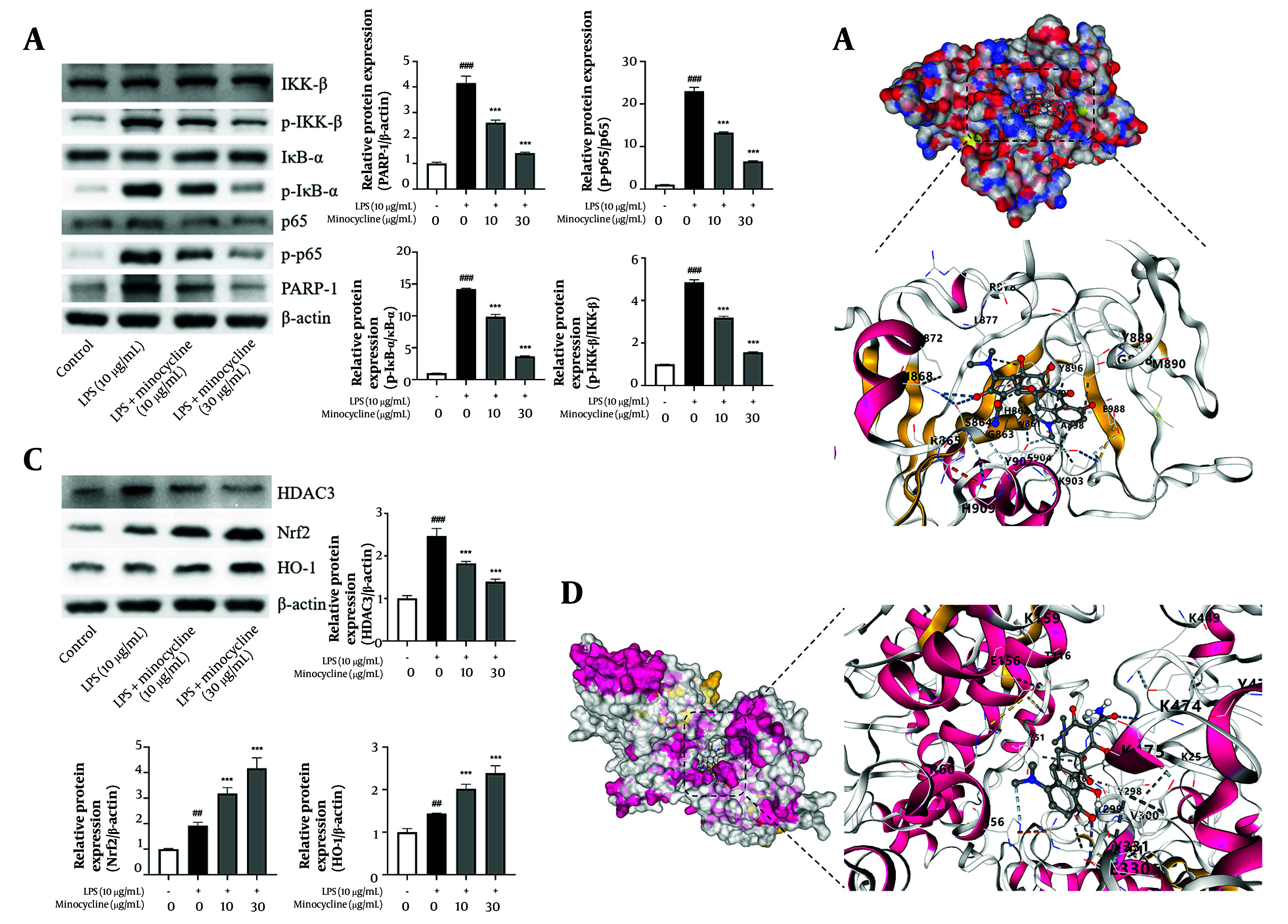
Minocycline reduces lipopolysaccharide-induced acute lung injury (ALI) through inactive poly (ADP-ribose) polymerase-1 (PARP-1) and histone deacetylase 3 (HDCA3) pathway. A and C, western blot analysis for PARP-1, p-p65, IKK-β, p-IKKβ, IκBα, p- inhibitor of kappa B (IκBα), nuclear factor erythroid 2-related factor 2 (Nrf2), heme oxygenase-1 (HO-1), and HDAC3 of lipopolysaccharide (LPS)-induced A549 cells; B and D, schematic diagram of molecular docking of minocycline with PARP-1 and HDAC3 [(n = 6) values are mean ± SD; ## P < 0.01, ### P < 0.001 vs. the control group, *** P < 0.001 vs. LPS-treated group.

Western blot analysis demonstrated that minocycline inhibited LPS-induced activation of PARP-1, and subsequently suppressed phosphorylation of IKKβ and IκBα, thereby preventing NF-κB p65 activation (Figure 4A). Molecular docking results showed that minocycline exhibited strong binding affinity to PARP-1 with a maximum binding energy of -8.1 kcal/mol (Figure 4B and Table 2). 

**Table 2.** Molecular Docking Results of Minocycline on Poly (ADP-ribose) Polymerase-1

CurPocket ID	Vina Score (kcal/mol)	Cavity Volume (Å3)	Center (x, y, z)	Docking Size (x, y, z)
**C1**	-8.1	233	-4, 37, 11	22, 22, 22
**C2**	-7.0	139	5, 51, 7	22, 22, 22
**C3**	-6.2	334	-3, 38, -3	22, 22, 22
**C4**	-6.1	194	15, 42, -1	22, 22, 22
**C5**	-5.7	515	18, 33, 16	22, 22, 22

Similarly, minocycline inhibited HDAC3 activation and enhanced the expression of downstream antioxidant factors Nrf2 and HO-1 (Figure 4C and D, Table 3, and Appendix 1 in Supplementary File). The docking simulations further confirmed favorable binding of minocycline to HDAC3, with a maximum binding energy of -8.7 kcal/mol.

**Table 3.** Molecular Docking Results of Minocycline on Histone Deacetylase 3

CurPocket ID	Vina Score (kcal/mol)	Cavity Volume (Å3)	Center (x, y, z)	Docking Size (x, y, z)
**C1**	-8.7	21618	30, 63, 22	35, 35, 35
**C2**	-7.2	373	23, 45, 35	22, 22, 22
**C3**	-6.5	366	44, 58, 36	22, 22, 22
**C4**	-6.3	345	42, 49, 0	22, 22, 22
**C5**	-6.0	789	27, 32, 25	22, 22, 22

These results collectively suggest that minocycline modulates inflammatory and oxidative stress pathways in ALI via inhibition of PARP-1 and HDAC3 signaling. Future comparative docking analyses with known inhibitors such as olaparib (PARP-1) and RGFP966 (HDAC3) will be necessary to fully elucidate minocycline’s therapeutic potential (exact P-values are listed in Appendix 5 in Supplementary File).

## 5. Discussion

Acute lung injury/ARDS are life-threatening lung diseases characterized by high mortality, often driven by acute lung inflammation (2). Excessive inflammation and oxidative damage of the lung play the major roles in mediating, amplifying, and perpetuating the lung injury process (19). Studies have shown that minocycline can inhibit neutrophil chemotaxis, expression of inflammatory factors, and remove oxidative factors during inflammation (14). Furthermore, minocycline can also inhibit apoptosis caused by inflammation by suppressing the formation of activated caspase3 and increase the expression of anti-apoptotic proteins (20). Our investigation has uncovered that minocycline also shows effective anti-inflammatory treatment for lung inflammation. The experiment data shows that minocycline can significantly inhibit lung inflammation and oxidative damage in mouse with LPS-induced acute lung injury, alleviate lung injury, reduce alveolar edema and protein exudation, and suppress neutrophil aggregation. Neutrophils are the first immune cells recruited to the site of injury in the early stage of ALI, but excessive activation of neutrophils can result in tissue damage. Myeloperoxidase is an enzyme located mainly in the primary granules of neutrophils, which reflects the adhesion and margination of neutrophils in the lung (17). Reduction of MPO activity predicts a favorable prognosis for ALI. So, minocycline maybe an excellent candidate for the treatment of ALI.

To delve deeper into the therapeutic mechanism of minocycline for ALI, the LPS-induced alveolar epithelial cells are employed for further study. The network of pro-inflammatory cytokines plays a vital role in lung injury. During ALI, TNF-α, IL-1β and IL-6 can directly damage vascular endothelial cells and alveolar epithelial cells, and further stimulate the secretion of other inflammatory factors to aggravate inflammatory injury (5, 6). Interleukin-8 and ICAM-1 have important effects on the activation and accumulation to pulmonary tissue of neutrophils during the development of ALI (7). Subsequently, the activation and chemotaxis of neutrophils further damage alveolar function. Our experimental results show that minocycline can effectively inhibit the expression of cytokines in inflammation damaged alveolar endothelial cells, which is advantageous in mitigating ALI tissue damage and neutrophil infiltration.

Oxidative stress is another critical factor in the pathophysiology of ALI. During ALI, expression of ROS and other oxidative damage factors, such as MDA, in alveolar epithelial cells may further exacerbate the inflammatory response, cell apoptosis, pulmonary edema, and tissue damage (21). Antioxidant enzymes, including SOD and GSH-Px, play a crucial role in eliminating reactive free radicals to maintain the balance between oxidative and anti-oxidative stress responses (22). Our results show that minocycline can effectively inhibit LPS-induced ROS production and MDA increasing, and increase the expression of antioxidant enzymes SOD and GSH-Px. We further investigate the molecular mechanisms involved in antioxidation defense. The antioxidant properties of minocycline may be attributed to the upregulation of the Nrf2/HO-1 pathway. The Nrf2/HO-1 signaling pathway serves as a critical mechanism for the body to counteract oxidative stress. Nuclear factor erythroid 2-related factor 2 is a master regulator of multiple antioxidant enzymes. When Nrf2 stimulated by external factors, it subsequently activation and entered the nuclear to enhance the expression of numerous downstream antioxidant enzymes (19). Heme oxygenase-1 is the most easily activated antioxidant enzyme by Nrf2.

Studies have shown that alveolar epithelial apoptosis is a key pathological feature in ALI, and is directly related to the degree of lung injury (23). Control alveolar epithelial apoptosis is a critical target for treatment. Our investigation substantiates the efficacy of minocycline in inhibiting apoptosis of alveolar epithelial cells and alleviates lung injury. Subsequent molecular mechanism studies have demonstrated that the inhibitory impact of minocycline on apoptosis is mainly mediated by the inhibition of cleaved-caspase 3 and proapoptotic Bax, as well as the activation of anti-apoptotic Bcl-2. Mitochondria is the center of multiple biological processes, such as cell apoptosis, redox homeostasis. The normal mitochondrial membrane potential is the prerequisite for mitochondrial function. Inflammatory injury will diminish the mitochondrial membrane potential, subsequently leading to apoptosis (24). The results of JC-1 showed that minocycline can effectively protect the mitochondria of alveolar epithelial cells damaged by inflammation. So, we speculate that the protective effect of minocycline on mitochondria may be ultimately inhibited the apoptosis.

Activation of the NF-κB p65 phosphorylation is an important signal for the regulation of cellular inflammation (25). Clinical studies have indicated that patients suffering from ALI exhibit a heightened degree of NF-κB activation. Inactivation of NF-κB pathway by pharmacological inhibition or gene modification decreased the expression of pro-inflammatory mediators and diminished the severity of ALI (26). Activation of NF-κB is a critical pathway for PARP-1 in the regulation of inflammation (9, 10). Activated PARP-1 can regulate the phosphorylation activation of IKKβ to p-IKKβ. The p-IKKβ will further phosphorylation activate IκBα to p-IκBα, and p-IκBα then dissociates from the NF-κB trimer (p65/p50/IκBα). The shedding of IκBα eventually allows NF-κB to be activated from the inhibited state. Subsequently, the p65 subunit of NF-κB is phosphorylated (p-p65) and entered the nucleus, ultimately regulating the expression of various target genes such as immunity and inflammation (27, 28). Consequently, the suppression of PARP-1 activation will ultimately regulate inflammation by influencing the NF-κB activation process. HDAC3 modulates a range of transcription factors, thereby influencing their regulation of the inflammatory response. Study has shown that RGFP966, a selective HDAC3 inhibitor, can reduce the levels of pro-inflammatory cytokines in a model of inflammatory lung disease (11). In addition, previous investigations have demonstrated HDAC3 exerts inhibitory effects on Nrf2 promoter activity (12). Inhibition of HDAC3 can delay oxidative stress by activating Nrf2. The western blot and molecular docking results show that minocycline can effectively inhibit the expression of PARP-1 and HDAC3 in LPS-induced A549 cells, which suggests a potential correlation with its protective effect against ALI.

### 5.1. Conclusions

In summary, our study demonstrates that minocycline confers significant protection against LPS-induced ALI. This protective effect is characterized by reduced lung injury, decreased pulmonary edema and protein leakage, inhibition of neutrophil infiltration, suppression of inflammatory cytokine expression, mitigation of oxidative stress, and prevention of epithelial cell apoptosis. Mechanistically, these effects may be mediated via inhibition of the PARP-1/NF-κB and HDAC3/Nrf2 signaling pathways. Taken together, our findings suggest that minocycline is a promising candidate for the treatment of ALI and warrants further investigation in clinical settings.

ijpr-24-1-161381-s001.pdf

## Data Availability

The data are available from the corresponding author upon reasonable request.
