# Supplementary material for: Minocycline Regulates PARP-1 and HDAC3 Pathways to Inhibit Inflammation and Oxidative Stress in LPS-Induced Acute Lung Injury
Source: Iran J Pharm Res. 2025 Jun 29;24(1):e161381. doi: 10.5812/ijpr-161381 (PMC12297026; doi:10.5812/ijpr-161381)
Supplement: ijpr-24-1-161381-s001.pdf [file ijpr-24-1-161381-s001.pdf]

1. Positive Control

To further verify the inhibitory effect of minocycline on HDAC3, we employed the HDAC3-selective inhibitor RGFP966 as a positive control to perform a comparison experiment. The western blot results are shown in Figure S1. RGFP966 (10 μg/mL) can significantly inhibit the expression of HDAC3 in LPS-induced A549 cells (p=0.000). The western blot results of minocycline have the same trend as those of RGFP966, which can significantly inhibit the expression of HDAC3 (p=0.000). However, the HDAC3-selective inhibitors exhibited stronger inhibition of HDAC3 than minocycline (p=0.000). According to the comparison results of positive drugs, minocycline has a definite inhibitory effect on HDAC3, and its drug potency is weaker than that of RGFP966.

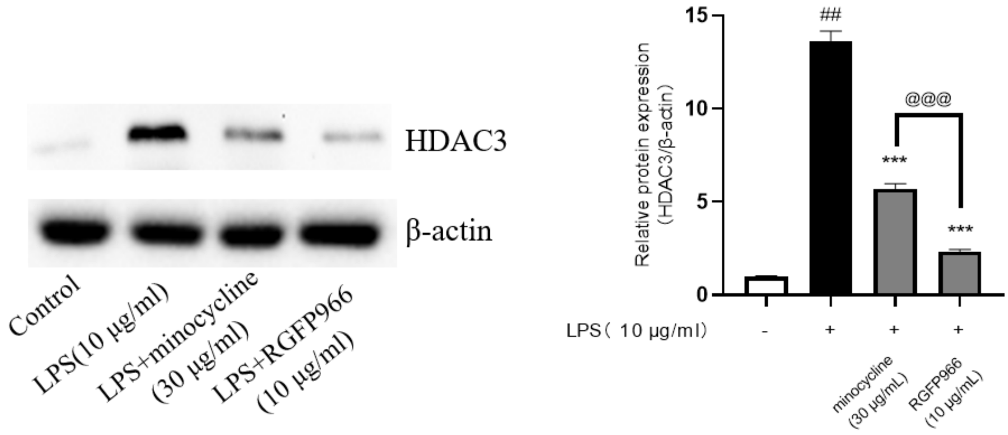

Appendix 1. Western blot analysis of the inhibitory effects of minocycline and RGFP966 on HDAC3. (n=6) Values are mean ± SD. ###P < 0.01 vs. the control group, ***p < 0.001 vs. LPS-treated group, @@@ p < 0.001 vs. minocycline (30 μg/mL) group.

2. Statistical Details

The exact p-values of all the experimental result statistics are shown as follows. The statistical details presented in the subsequent tables (**Table S1-S4**) accurately correspond with each image depicted in the article.

Appendix 2. **The exact *p*-values of all the results in Figure 1**

LPS(40μg/mL)	Minocycline (mg/kg)	*p*-values										
		TNF-α in BALF	IL-1β in BALF	IL-6 in BALF	MDA	SOD content	Lung injury score	W/D ratio of lung	Relative protein in BALF	Total cells in BALF	Neutrophils in BALF	MPO activity
-	0	-	-	-	-	-	-	-	-	-	-	-
+	0	0.000	0.000	0.000	0.000	0.000	0.000	0.000	0.000	0.000	0.000	0.000
+	10	0.000	0.000	0.000	0.000	0.000	0.000	0.001	0.000	0.000	0.000	0.000
+	30	0.000	0.000	0.000	0.000	0.000	0.000	0.000	0.000	0.000	0.000	0.000

Appendix 3. **The exact *p*-values of all the results in Figure 2**

LPS(40μg/mL)	Minocycline (mg/kg)	*p*-values								
		Relative TNF-α mRNA expression	Relative IL-1β mRNA expression	Relative IL-6 mRNA expression	Relative IL-8 mRNA expression	Relative ICAM-1 mRNA expression	Relative ROS levels	GSH-Px	MDA	SOD
-	0	-	-	-	-	-	-	-	-	-
+	0	0.000	0.000	0.000	0.000	0.000	0.000	0.000	0.000	0.000
+	10	0.000	0.000	0.000	0.000	0.000	0.001	0.000	0.000	0.000
+	30	0.000	0.000	0.000	0.000	0.000	0.000	0.000	0.000	0.000

Appendix 4. **The exact *p*-values of all the results in Figure 3**

LPS(40μg/mL)	Minocycline (mg/kg)	*p*-values				
		apoptotic rate	Relative intensity of fluorescence	Bcl-2	Bax	Cleaved-caspase 3
-	0	-	-	-	-	-
+	0	0.000	0.000	0.000	0.000	0.000
+	10	0.000	0.000	0.027	0.000	0.000
+	30	0.000	0.000	0.000	0.000	0.000

Appendix 5.**The exact *p*-values of all the results in Figure 4**

LPS(40μg/mL)	Minocycline (mg/kg)	*p*-values						
		PARP-1	p-p65/p65	p-IκB-α/ IκB-α	p-IKK-β/ IKK-β	HDAC3	Nrf2	HO-1
-	0	-	-	-	-	-	-	-
+	0	0.000	0.000	0.000	0.000	0.000	0.002	0.001
+	10	0.000	0.000	0.000	0.000	0.000	0.000	0.000
+	30	0.000	0.000	0.000	0.000	0.000	0.000	0.000
